# *Eugenia aurata* and *Eugenia punicifolia HBK* inhibit inflammatory response by reducing neutrophil adhesion, degranulation and NET release

**DOI:** 10.1186/s12906-016-1375-7

**Published:** 2016-10-22

**Authors:** Mírian Feliciano Costa, Tais Iara Jesus, Bruno Rafael Pereira Lopes, Célio Fernando Figueiredo Angolini, Abner Montagnolli, Lorraine de Paula Gomes, Gabriela Sterle Pereira, Ana Lucia Tasca Gois Ruiz, João Ernesto Carvalho, Marcos Nogueira Eberlin, Catarina dos Santos, Karina Alves Toledo

**Affiliations:** 1 Departamento de Ciências Biológicas, Faculdade de Ciências e Letras, Universidade Estadual Paulista -UNESP, Av Dom Antônio, 2100, Parque Universitário, ZIP 19806-900 Assis, SP Brazil; 2 ThoMSon Laboratório de Espectrometria de Massas, Instituto de Química, Universidade Estadual de Campinas (UNICAMP), Campinas, 13083-970 Brazil; 3 Centro Pluridisciplinar de Pesquisas Químicas, Biológicas e Agrícolas, UNICAMP, CP 6171, CEP 13083-970 Paulínia, SP Brazil; 4 Faculdade de Ciências Farmacêuticas, Universidade Estadual de Campinas (UNICAMP), P.O. Box 859, Campinas, 13083-859 Brazil

**Keywords:** Inflammation, Neutrophils, *Eugenia aurata*, *Eugenia punicifolia* (HBK), Adhesion, Elastase

## Abstract

**Background:**

*Eugenia* spp. are used in popular medicine in the treatment of pain, diabetes, intestinal disorders and cough. The aim of the work is to evaluate, ex vivo and in vivo, the anti-inflammatory activity of the hydroethanolic extracts of the leaves of *Eugenia aurata* (EA) and *Eugenia punicifolia* HBK (EP) upon neutrophils.

**Methods:**

Ex vivo, isolated human neutrophils were sensitized by *Eugenia* extracts (0.1–1000 μg/mL) and stimulated by PMA. In these conditions, different neutrophil activities related to inflammatory process were measured: adhesion, degranulation and NET release. Neutrophil viability and tumor line cells were monitored. In vivo, neutrophil influx was evaluated by peritonitis model performed in mice pretreated with different concentrations of *Eugenia* extracts. Phytochemical profile was assessed by mass spectrometry.

**Results:**

Ex vivo, EA and EP (1000 μg/mL) reduced cell adhesion and degranulation, respectively. NET release was inhibited by EA and EP. Anti-inflammatory activities occurred in the absence of cytotoxicity. In vivo, both EA as EP inhibited neutrophil migration. The phytochemical profile revealed that EA contains myricitrin, rutin, quinic acid and quercetin derivatives. EP presents gallic acid, quercetin derivatives, syringic acid, ellagic acid, monogalloyl-glucose, glycosyringic acid, mudanoside B, HHDP glucose isomer and digalloylglucose isomer. EA and EP inhibit neutrophil migration by different pathways.

**Conclusion:**

Different chemical compositions may explain the anti-inflammatory effects described herein for EA and EP. Both extracts inhibit NET release but only EA reduces cell adhesion whereas EP decreases elastase secretion. This work contributes to the elucidation of cellular mechanisms related to the anti-inflammatory activity for leaves of *E. aurata* and *E. punicifolia* HBK*.*

## Background

Inflammation is a process that includes a complex immune response, which occurs in several steps and may be caused by chemical, physical, microbiological and immunological stimuli. It involves leukocyte recruitment where the first leukocytes to be recruited and act on the inflamed tissue are neutrophils. Neutrophils have been considered a target for pharmacological intervention given their abilities to kill microorganisms, to begin and amplify the inflammatory process. Neutrophil recruitment and inflammatory activities require a complex sequence of events, including cell adhesion, degranulation, and more recently, neutrophil extracellular traps (NET) release [1]. The control of the inflammatory process is critical because of the associated risks: tissue damage, loss of organ performance and failure.

*Eugenia* genus with over 500 species, of which about 400 are in Brazil, assumes prominence in popular medicine, mainly for their anti-inflammatory activities in the treatment of wounds and infections [2, 3]. Flavonoids, tannins, terpenoids and essentials oils were isolated from this genus [4, 5]. Different crude extracts of *Eugenia* show several medicinal effects, such as anti-inflammatory [6], antifungal [7], neurological [8], antimicrobial [9], among others. Leaves of *Eugenia punicifolia* are popularly used to treat inflammation [10], diabetes [6, 10], fever and flu [11, 12]. *E. aurata* is an endangered species [13] with low studies in the literature and, by analogy, there is a need of registering its effects on inflammatory processes.

Although leaves of *Eugenia* species are widely used in popular medicine for inflammatory diseases, efficacy of cellular and molecular mechanisms remains elusive. Our aim was to evaluate the cellular mechanisms involved in the anti-inflammatory activity of *Eugenia aurata* and *Eugenia punicifolia*. For this purpose, ex vivo assays were performed and the anti-inflammatory activity was confirmed using in vivo assays.

## Methods

### Collection and preparation of extract

Leaves of *E. punicifolia* and *E. aurata* were collected in December (2009) in Assis (Instituto Florestal e Estações Experimentais – Floresta Estadual de Assis) at the point (UTM 0561750 L/O 7500935 (+/- 3 m) - 0559055 L/O 7499970 (+/- 4 m)), São Paulo State, Brazil. Dr. Antônio C.G. Melo identified the specimen and voucher specimen (n° 43.522 and 43520, respectively) were deposited in Herbarium D. Bento Pickel for future reference. The extract has been prepared with 10 g of plant material (dried and triturated leaves) and 100 ml of solvent (Ethanol:H_2_O 70:30 v/v). The extract solution was obtained by 2 h dynamic maceration at room temperature (25 ± 2 °C), followed by filtration. Remaining extract residue filtration was carried twice by the same procedure. Subsequently, the solution was dried at 40 °C temperature with a rotary evaporator, obtaining 45 % and 7 % hydroethanolic extract solutions from *E. punicifolia* (HEEP) and *E. aurata* (HEEA), respectively. The hydroethanolic extract fraction soluble in Phosphate Buffer Solution (PBS; 137 mM NaCl, 2.7 mM KCl, 10 mM Na_2_HPO_4_, 1.8 mM KH_2_PO_4_) was evaluated in all bioassays (*Eugenia aurata* = EA; *Eugenia punicifolia* = EP).

### Animals

Swiss male mice, weighing on average 40 grams, were kept in controlled temperature rooms (23–25 °C) with access to food and water. All animal care and experimental procedures were performed in accordance with the internationally accepted principles for laboratory animal use [14].

### Human neutrophils isolation

Human neutrophils were isolated and prepared according to previous methods described [15, 16]. Cells were suspended in Hank´s balanced salt solution (HANKS) (Sigma, St. Louis, MO, USA) containing 0.1 % gelatin (w/v) (HANKS-gel) with over 90 % viability as determined by the Trypan blue (Sigma) exclusion test, and 90–95 % of cells were found to be neutrophils.

### Cell viability (MTT assay)

Cytotoxicity was evaluated by the colorimetric method of MTT (3-(4,5-dimethylthiazol-2-yl) 2,5-Diphenyl Tetrazolium bromide) (Sigma), which consists of indirectly measuring of cell viability by mitochondrial enzyme activity of living cells. Human neutrophils (2 x 10^5^ / well) seeded into 96 well culture plates were incubated with different concentrations of *Eugenia* spp. during 1 h at 37 °C. Subsequently, MTT (1 mg/mL) was added to each well and incubated at 37 °C for 4 h. After incubation, formazan crystals were diluted by addition of Dimethyl Sulfoxide (DMSO, Sigma) and the optical density (O.D.) of samples measured in a spectrophotometer at 570 nm. Neutrophils incubated either with RPMI-1640 (Sigma) or 50 μM H_2_O_2_ [17] were used as negative and positive control (100 % viable) to cell death, respectively.

### Cell adhesion

Cell adhesion assays were performed in 96 well micro plates. Human neutrophils (4 x 10^5^) suspended in RPMI medium (Sigma) plus 5 % Fetal Bovine Serum (FBS) (Vitrocell, Campinas, SP, Brazil) were added to wells of a micro plate containing different concentrations of *Eugenia* spp. After 15 min, cells were then stimulated by Phorbol Myristate Acetate (PMA 25nM) (Sigma) for 1 h at 37 °C. Non-adherent cells were removed and adherent cells were made evident via a colorimetric test with Bicinchoninic Acid (BCA; Pierce). Sample absorbance was measured in a Multiskan FC (Thermo Scientific, Waltham, MA, USA) reader at 560 nm.

### Elastase activity

Elastase enzyme activity upon degranulation was assessed as follows: Neutrophils (2 x 10^5^) suspended in Hank's solution were incubated for 30 min in the presence of different *Eugenia* spp. concentrations then stimulated with PMA (25nM) for 3 h at 37 °C. Succeeding incubation, neutrophils were centrifuged (437 x g, 5 min) and the resulting supernatants incubated in 1 mM elastase substrate (N-Methoxysuccinyl-Ala-Ala-Pro-Val p-nitroanilide) (Sigma) for 30 min. After incubation, color reaction was measured at 405 nm on microplate reader Multiskan FC (Thermo Scientific). Several concentrations of purified elastase enzyme from human neutrophils (EMD Chemicals Inc., Billerica, MA, USA) were used as standards.

### Neutrophil Extracellular Traps (NETs) release

Human neutrophils (2 x 10^5^) were incubated with different concentrations of *Eugenia* spp. during 30 min and then stimulated with PMA (50 nM) for 4 h at 37 °C. NETs generated by activated neutrophils were digested with 500 mU/mL micrococcal nuclease (MNase, Worthington Biochemical Corp.) [18]. The nuclease activity was ceased by 5 mM Ethylene Diamine Tetra Acetic Acid (EDTA) and the culture supernatant collected and stored at 4 °C until the moment of quantification. NETs were quantified using the PicoGreen dsDNA kit (Invitrogen) according to the manufacturer's recommendations.

### Peritonitis model (in vivo)

Mice received 1 mL of 3 % Thioglycolate injected intraperitoneally one hour after plant extract subcutaneous administration (3-300 mg/kg) [19, 20]. Six hours later, mice were euthanized by cervical dislocation. The cells were immediately harvested with 5 mL PBS containing heparin (5 IU/mL). Total counts of harvested cells were performed in a Neubauer chamber. Differential counts were made on smears stained using Panoptic Fast Stain (LaborClin, Siqueira Campos, PR, Brazil). The results were reported as the number of neutrophils per mL of cavity wash. The control groups animals received: (1) subcutaneous and intraperitoneal PBS injection; (2) subcutaneous PBS and intraperitoneal Thioglycolate injection; (3) subcutaneous Dexamethasone (0.5 mg/kg) and intraperitoneal Thioglycolate injection.

### Evaluation of antiproliferative activity in vitro

Antiproliferative activity was tested against cell lines: UACC-62 (melanoma); MCF-7 (mammary); NCI-ADR/RES (drug resistant ovary); 786-0 (kidney); NCI-H460 (lung); PC-3 (prostate); OVCAR-3 (ovary); HT-29 (colon), K562 (leukemia) and VERO (African green monkey kidney cell line). Stock cultures were grown in a medium containing 5 mL RPMI 1640 (Sigma) supplemented with 5 % fetal bovine serum. Gentamicin (50 mg/mL) was added to experimental cultures. Cells in 96 well plates (100 μL cells/well) were exposed to sample concentrations of DMSO/RPMI (0.25, 2.5, 25, and 250 μg/mL) at 37 °C, 5 % CO_2_ in air for 48 h. Next, cells were fixed with 50 % trichloroacetic acid and cell proliferation was determined employing sulforhodamine B assay at 540 nm [21]. Using the concentration–response curve for each cell line, TGI (concentration that produces total growth inhibition or cytostatic effect) was determined through non-linear regression analysis, utilizing software ORIGIN 8.5 (OriginLab Corporation) [22].

### Phenolic content

Phenolic content was performed as previously described [23] with minor adaptations. Briefly, 2.5 mL Folin-Ciocalteau 10 % (v/v) and 2.0 mL 4 % (m/v) sodium carbonate were added to a 0.5 mL extract in ethanol solution (1 mg/mL). After a 2 h incubation in the dark, at room temperature, absorbance was measured at 750 nm and results were expressed as equivalent milligrams of gallic acid per gram of sample. All tests were performed in triplicate.

### Flavonoid content

Flavonoid content was performed as previously described [23] with minor adaptations. Briefly, 1.5 mL ethanol, 0.1 mL potassium acetate (1 M) and 2.8 mL distilled water were added to a 0.5 mL extract in ethanol solution (1 mg/mL). After 30 min incubating in the dark at room temperature, absorbance was measured at 425 nm and results were expressed as equivalent milligrams of quercetin acid per gram of sample. All tests were performed in triplicate.

### ESI-MS^n^ analysis

The mass spectrometry experiments were performed on a 6550 iFunnel Q-TOF (Agilent Technologies). The studied matrix was analyzed by Dual Agilent Jet Stream ESI (Dual-AJS-ESI) (ESI) and fragmented in the MS/MS collision cell. The negative mode was selected for the generation and analysis of first order mass spectra (MS) and the remaining multistage experiments under the following conditions: Gas Temp at 290 °C, Drying Gas flow at 11 Lmin^−1^, Nebulizer at 45 psi, Sheath gas temp at 350 °C, Sheath gas flow 12 Lmin^−1^, VCap 3000, Nozzle voltage 320 V, Fragmentor 100 V, OCT 1 RFVpp 750 V, and collision energy 35 V Agilent MassHunter Qualitative Analysis software version B.06.00 used for data acquisition and processing.

### Statistical analysis

Experimental data was evaluated by variance analysis (one-way ANOVA) followed by Bonferroni test. A significance level of 5 % was adopted. All assays were performed in triplicate at least in three independent assays.

## Results and discussion

Inflammatory process involves cellular and molecular events that begin with neutrophil recruitment. This process is commonly separated in four steps: rolling, adhesion, transmigration and degranulation. The rolling is mediated by the interaction between neutrophil selectins (selectin- L) and endothelium selectin (selectins P and E). Sequentially, the adhesion happens by the interaction between endothelium and neutrophil integrins as well as by mobilization of neutrophil secretory vesicles. After that, the transmigration - or diapedesis – also occurs under effect of integrins interactions and counting on the help of release of neutrophil tertiary granules to digest endothelium basal membrane. Finally, the proteins released from neutrophil secondary/primary granules can be associated with DNA and oxidative enzymes, also called NETs. The release of NETs improves microbe capture, as well as increases phagocytosis efficiency [1, 24].

The cellular mechanisms related to the anti-inflammatory activity of *E. punicifolia* (EP) and *E. aurata* (EA) were evaluated. Therefore, some neutrophil functions were analysed ex vivo and others in vivo because neutrophils are the first inflammatory cells to be recruited to the damaged tissue. Both *Eugenia* extracts were able to inhibit neutrophils responses, by different pathways, under clear evidence of no toxicity for the cells.

**Fig. 1:**
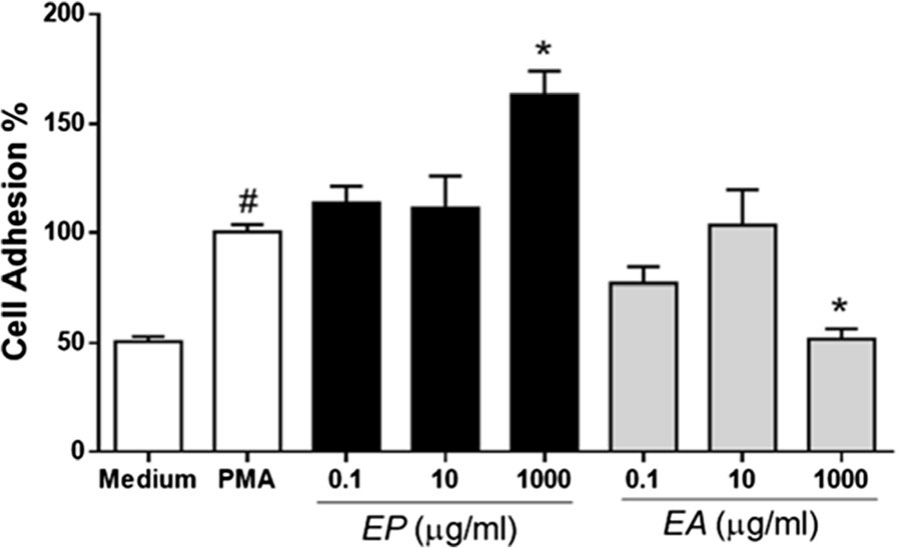
*Eugenia aurata* (EA) inhibits human neutrophil adhesion. Human neutrophils (4x10^5^) were pretreated with different concentrations of EA or EP (15 min) and stimulated (1 h) to adhesion by PMA (25nM). Neutrophils incubated with RPMI alone were used as negative control. Data are shown as cell adhesion (%) ± S.D. where PMA is 100 %. *p* <0.05 when compared to PMA (*) or medium (#) control

Ex vivo assays aimed the evaluation of the role of *EA* and *EP* in adhesion, elastase secretion and NET release. In the first step, adhesion, neutrophils incubated with 25 nM PMA and adhered to the culture plate were considered as 100 % adhesion (Fig. 1). Neutrophils incubated only with culture medium RPMI-1640 showed basal adhesion rate (~50 %). Pretreatment of neutrophils with several EP concentrations did not alter their ability to adhere under PMA stimulus. On the other hand, pretreatment with EA 1000 μg/mL significantly reduced cell adhesion. EP (1000 μg/mL) was the only concentration able to induce a weak neutrophil adhesion when neutrophils were incubated with extracts alone (*data not shown*), although this effect has not been statistically significant.

**Fig. 2:**
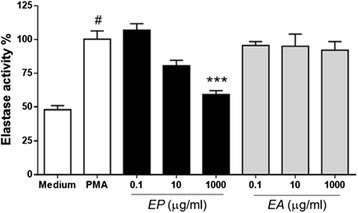
*Eugenia punicifolia* (EP) inhibits human neutrophil elastase secretion. Human neutrophils (4x10^5^) were pretreated with different concentrations of EA or EP (30 min) and stimulated to degranulation by PMA (25nM) for 3 h. Neutrophils incubated with only Hank´s (medium) were used as negative control. Data shown Elastase activity (%) ± S.D. where PMA is 100 %. *p* <0.01 when compared to PMA (**) or medium (##) control

The second step was to evaluate elastase secretion, indirectly assessed through elastase enzyme activity. Supernatants of cultures of neutrophils stimulated by PMA (25nM) and previously sensitized by plant extracts were analyzed. The release of elastase induced by PMA was considered as100%. Neutrophils incubated with medium alone showed significant reduction (Fig. 2). The presence of EA did not alter elastase secretion at any tested concentration . However, EP 1000 μg/mL significantly reduced PMA induced elastase secretion. None of the extract concentrations in test was able to induce elastase secretion (*data not shown*).

**Fig. 3:**
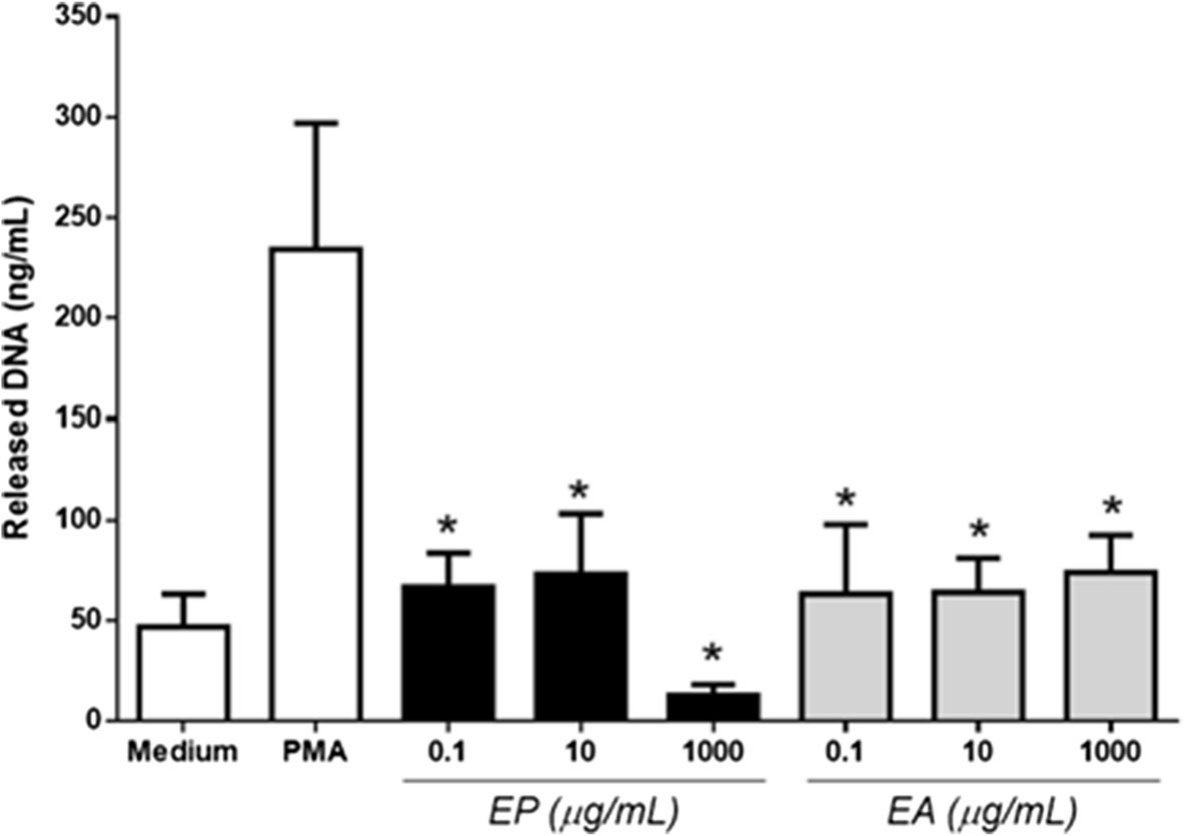
*Eugenia* spp. extracts inhibit NET release induced by PMA. Human neutrophils (4x105) were pretreated with different concentrations of *E. aurata* (*EA*) or *E.punicifolia* (*EP*) (0.1-1000 μg/mL) and stimulated to NET release for 4 h by 50nM PMA. Neutrophils incubated with PMA or Hank´s (medium) alone were used as positive and negative control, respectively. Data shown as released DNA (ng/mL) ± S.D. *p* <0.01 when compared to PMA (*) or medium (#) control

Next inflammatory event evaluated was the release of NETs. The presence of both plant extracts studied here inhibited significantly the release of NETs induced by PMA (50nM) for all tested concentrations (0.1–1000 μg/mL) (Fig. 3). The positive control (50nM PMA) induced DNA release of 230 ng/ml while negative control (Medium) showed ~50 ng/mL. The inhibition of this event may have a suppressive effect on inflammation, activation of neutrophils and capture/elimination of pathogens by decreasing the inflammatory stimulus that comes from genetic material released. Furthermore, the presence of extracellular DNA (NETs) has been appointed as a direct source of stimulus to inflammatory and autoimmune diseases [1].

**Fig. 4:**
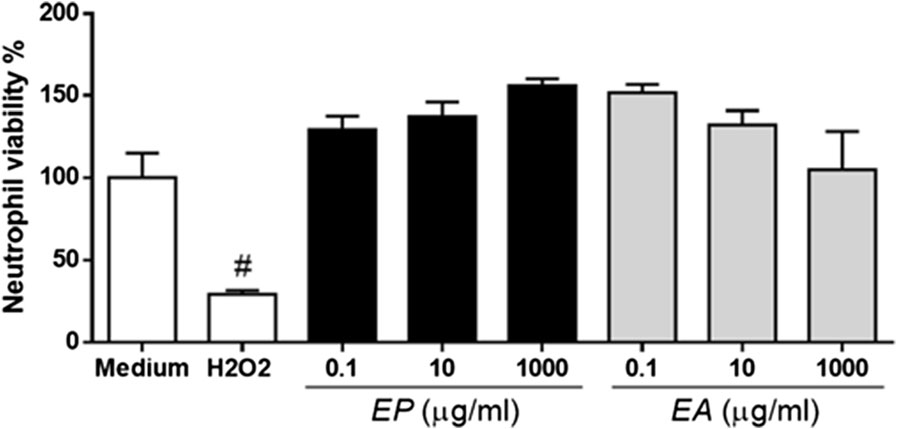
Neutrophils viability is not affected by *Eugenia aurata* (EA) or *E. punicifolia* (EP). Human neutrophils (4x10^5^) were incubated with different concentrations of EA or EP. At the end of incubation, cell viability was measured by MTT assay. Neutrophils incubated with RPMI (medium) alone or 50 μM H_2_O_2_ (Hydrogen Peroxide) were used as negative and positive control, respectively. Data shown as Neutrophil viability (%) ± S.D. *p* <0.01 when compared to Medium (*)

Neutrophil viability was monitored during all ex vivo assays. This is an important factor once some inhibitory effects could be related to cytotoxicity. The viability was assessed by MTT assay. The data revealed that the extracts, evaluated in different concentrations (0.1–1000 ug/mL), did not reduce neutrophil viability when these cells were sensitized either with EP or with EA (Fig. 4). As positive and negative controls, H_2_O_2_ and RPMI medium were used. H_2_O_2_ (50 μM) reduced 70 % of cell viability.

Since no cytotoxic effects were observed in leukocytes, other cytotoxicity assays were performed with *E. punicifolia* hydroethanolic extract solutions (HEEP) as well as with *E. aurata* hydroethanolic extract solutions (HEEA), using tumor and not tumor cell lines. As others [25], HEEP and HEEA (mean TGI > 141 μg/mL and 198 μg/mL, respectively) did not exhibit a cytotoxicity against all tested cell lines. HEEP presented a moderate selective activity effect against cell line K562 (leukemia, 12.9 ± 7.19 μg/mL) and weak for MCF-7 (mammary, 39.0 ± 5.80 μg/mL). For all the analyzed lineages, including normal cell VERO (Green monkey kidney), HEEP was inactive (Table 1). HEEA was inactive for the tested lineages, including normal cell VERO (Green monkey kidney).

**Table 1:** TGI values (Total Growth Inhibition, μg/mL) of *Eugenia punicifolia* and *E. aurata* hydroethanolic leaf extracts

Tested material	Cell lines^a^									
	u	M	a	7	4	p	o	h	k	V
Doxorubicin^b^		3.30	6.60	2.67	0.90	5.85	2.95	3.90	8.43	8.43
HEEP^c^	>250	39.0 ± 5.80	120.0 ± 0.97	>250	209.7 ± 3.26	47.6 ± 13,3	105 ± 53,7	124.7 ± 0.99	12.9 ± 7.19	>250
HEEA^c^	*	>250	>250	240 ± 0.14	78.5 ± 21.7	229 ± 64	*	>250	34.2 ± 9.7	>250

^a^u = UACC (melanoma); m = MCF-7 (mammary); a = NCI-ADR/RES (drug resistant ovary); 7 = 786-0 (kidney); 4 = NCI-H460 (lung); p = PC-3 (prostate); o = OVCAR-3 (ovary); h = HT-29 (colon) V = VERO (Green monkey kidney). ^b^Positive Control.^c^HEEP and HEEA – Ethanol:water 70:30 v/v extract. ^*^not tested

According to ex vivo assays, *E.aurata* and *E.punicifolia* inhibit neutrophil functions in the absence of cell death. Based on these results, in vivo tests were performed. The in vivo experiment comprises a greater complexity of events when compared to in vitro and ex vivo experiments, in which experimental conditions are better monitored. Therefore, the results obtained using in vivo analyses are closer to the real scenario.

**Fig. 5:**
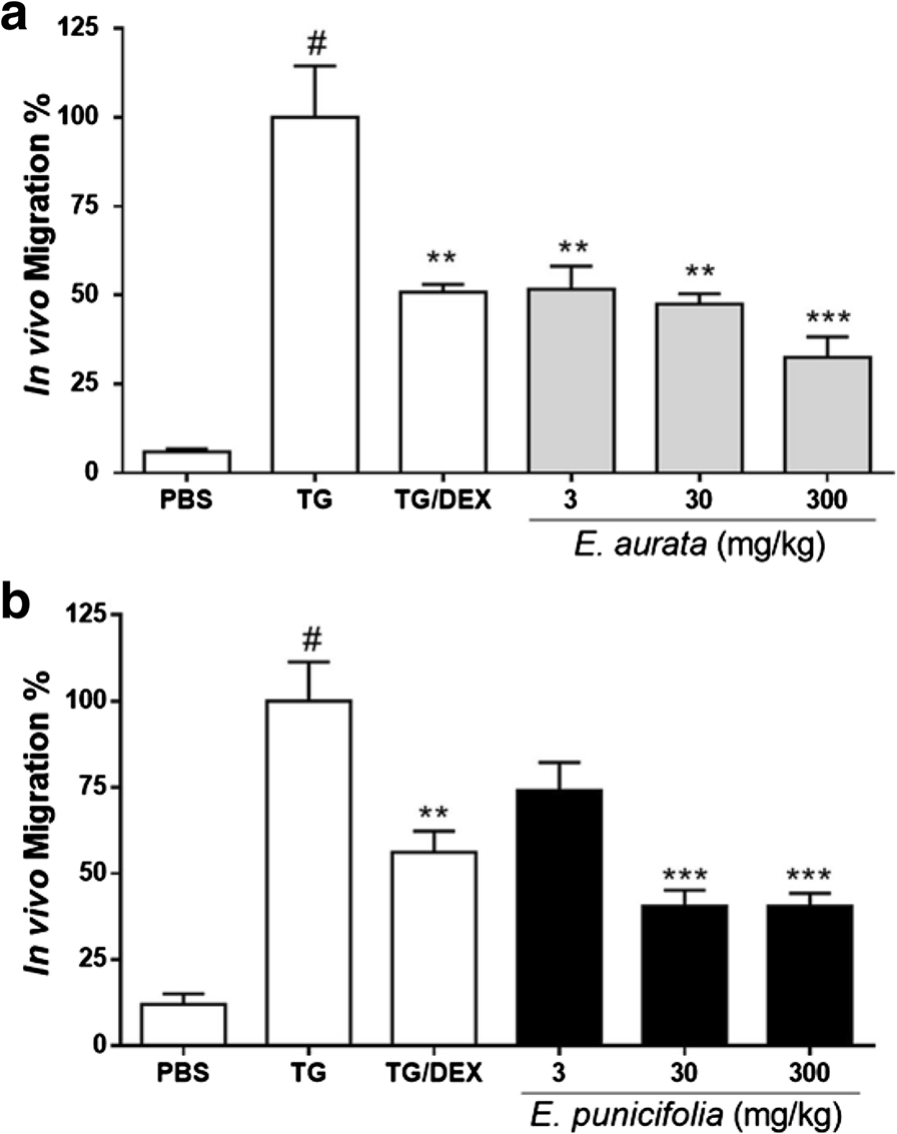
*Eugenia* extracts inhibit in vivo neutrophil migration. Swiss male mice previously injected (s.c.) with *Eugenia aurata* (EA; panel **a**) or *E. punicifolia* (EP; panel **b**), received i.p. injection of 3 % Thioglycolate (TG). Cellular migration was allowed for six hours when the peritoneal lavage fluid was collected and subjected to total and differential cell count. PBS group received = PBS (s.c. and i.p.); TG group received = PBS (s.c.) and TG (i.p.); TG/DEX group received = DEX (s.c.) and TG (i.p.). Data shown as in vivo migration (%) ± SD. (n = 5). # p <0.01 when compared to PBS; ** *p* <0.01 and *** *p* <0.001 when compared to TG

Acute peritonitis model induced by Thioglycolate in mice was assessed in order to evaluate *Eugenia* anti-inflammatory activity. Both extracts showed anti-inflammatory effect by inhibiting neutrophil influx. No animal showed symptoms of toxicity or even death. Mice received a subcutaneous injection of extract, and 1 h later were administered with 3 % Thioglycolate (TG) intraperitoneally. When mice are assayed in a peritonitis model, a 6 h period is necessary to reach maximum acute neutrophil recruitment. In vivo cell migration analysis showed that subcutaneous injection of different extracts concentrations significantly reduced cell influx into the peritoneal cavity (Fig. 5). EP showed anti-inflammatory activity at concentrations of 30 and 300 mg/mL (Fig. 5a). As for EA, the anti-inflammatory activity was observed for all the tested concentrations (Fig. 5b). As negative control, extracts were s.c. injected and PBS was i.p. injected. As a result, neutrophil migration did not occur. Either for EA as for EP, the anti-inflammatory activity was comparable to the effect of dexamethasone (DEX), a potent anti-inflammatory drug in clinical use.

Differences in chemical composition between *E. aurata* and *E. punicifolia* HBK may explain the anti-inflammatory effects described herein. Both extracts inhibit NET release but only EA reduces cell adhesion whereas EP decreases elastase secretion.

The control of accute/chronic inflammatory processes as rheumatoid arthrite, ashtma, vaculitis among others like diabetes is relevant, once such processes may be related to the damage caused by the release of free radicals [26]. Moreover, neutrophil activation is largely dependent on the generation of reactive oxygen species (ROS) that are known to be inhibited by antioxidant compounds, as catechin and rutin, found abundantly in plant extracts [27]. Selected phenolic compounds, as diosmin and hesperidin, decrease the adhesion of inflammatory cells to the endothelium [28], whereas others can inhibit degranulation of neutrophils without affecting superoxide production [29].

*E. punicifolia* showed phenolic compounds concentrations of 74.86 ± 0.02 mg gallic acid/g extract and *E. aurata* 57.93 ± 0.05 mg gallic acid/g extract. Flavonoid content found was 32.00 ± 0.02 mg quercetin/g extract and 15.78 ± 0.01 mg quercetin/g extract, respectively.

Magina et al. [30] described, respectively, for *Eugenia brasiliensis*, *E.umbelliflora* and *E.beaurepaireana* hydroethanolic leaves extracts (70 %): 162.6 ± 3.3, 138.0 ± 2.7 to 128.1 ± 2.9 mg gallic acid/g. *E. aurata* and *E. punicifolia* showed lower phenol levels when compared to species studied by Magina et al. [30]. Although phenolic content found is lower than expected for *E. punicifolia,* flavonoid content approached similar levels to previous studies from Magina and collaborators [30]: *E. brasiliensis*, *E. umbelliflora* and *E.beaurepaireana* showed, respectively, 14.4 ± 1.1, 31.2 ± 1.7 and 10.4 ± 1.1 mg quercetin/g extract.

In addition, after assessing the phenolic and flavonoid contents, an ESI-MS was performed, in order to correlate the main secondary metabolites found in HEEP and in HEEA with their biological activity without further chromatographic separations, since a preliminary HPLC HEEP analysis was already published by our group [31]. Gallic acid derivatives, flavonols, glycosides and procyanidins were the most common phenolic compounds in fruits and leaves of the Myrtaceae family [32]. Database search showed no prior HEEA research published to the moment.

The most characteristic corresponding molecular formulas of HEEP and HEEA, their fractions, MS/MS fragments are shown in Table 2. All peaks found were tentatively assigned based on their accurate masses and MS/MS patterns. The peak at *m*/*z* 169 was assigned to gallic acid and confirmed by fragmentation of m/z 125 [M-44-H]^**−**^ because of CO_2_^−^ loss [33]. The ion of *m/z* 191 represents quinic acid, frequently found in higher plant as major compounds in the leaves [34] showed fragments of *m/z* 173 after H_2_O loss [35]. The peak at *m/z* 359 was characterized as glycosyringic acid [36]. 3-Feruloylquinic acid (peak of *m/z* 367) was characterized according Fang et al. [37] and with fragment *m/z* 173 and diagnostic peak *m/z* 193 (hydroxymethoxycinnamoyl moiety). The peak at *m/z* 463 was characterized as mudanoside with fragments *m/z* 301 ([M-H-162]^−^) lost glucose group [38].

**Table 2:** Phenolic compounds tentatively identified of *Eugenia sp.* leaf extracts

Formula [M-H]^−^	Theoretical mass	Experimental mass [M – H]^−^ *m/z*	Δm (ppm)	MS/MS fragments *m/z*	Compound identification	HEEP HEEA	EA EP
C_7_H_5_O_5_	169,0142	169,0148	−1,49	151,125	gallic acid	HEEP	Ep
C_7_H_10_O_6_	191,0561	191,0561	0,06	173,134	quinic acid	HEEA	Ea
C_7_H_11_O_6_	197,0458	197,0461	−1,28	169, 140, 124	syringic acid	HEEP	Ep
C_14_H_5_O_8_	300,9990	300,9996	−1,69	284, 257, 229, 185	ellagic acid	HEEP HEEA	Ep
C_13_H_15_O_10_	331,0671	331,0670	−0,39	271, 211, 169	monogalloyl-glucose	HEEP HEEA	Ep
C_15_H_20_O_10_	359,0984	359,0979	1,31	271,169	Glycosyringic acid	HEEP	Ep
C_17_H_19_O_9_	367,1035	367,1050	0,29	326,193,173,134	3-Feruloylquinic acid	HEEA	
C_19_H_13_O_12_	433,0412	433,0423	−2,11	300,169, 125	Ellagic acid xyloside	HEEP	
C_20_H_17_O_11_	433,0776	433,0768	2,52	300,271,169	Quercetin-3-O-α − arabinopyranoside	HEEP	Ep
C_21_H_19_O_11_	447,0933	447,0936	−0,7	301, 271,151	Quercetin-3-O-β − rhamnose	HEEP HEEA	Ep Ea
C_21_H_19_O_12_	463,0880	463,0882	0,43	317, 271, 179	myricitrin	HEEA HEEP	Ea
C_18_ H_23_O_14_	463,1166	463,1086	1,19	301, 169	Mudanoside B	HEEP	Ep
C_20_H_17_O_14_	481,0624	481,0642	−3,78	447, 301, 275, 211,169	HHDP glucose isomer	HEEP	Ep
C_23_H_31_O_11_	483,1872	483,1858	2,16	447, 331,169	Digalloylglucose isomer	HEEP	Ep
C_27_H_30_O_15_	593,1502	593,1547	1,67	415, 341, 284,103	rutinosylkaempferol	HEEA	
C_27_H_19_O_14_	609,1480	609,1461	−3,1	511, 300, 151	Rutin	HEEA	Ea
C_39_H_19_O_8_	615,1087	615,1086	−0,1	463, 301, 241, 169	Quercetin galloylhexoside isomer	HEEP	Ea Ep
C_34_H_24_O_22_	783,0686	783,0681	0,7	481, 381, 275	bis HHDP-glucose isomer	HEEP	

Ep = *E. punicifolia*; Ea = *E. aurata* (HEEP and HEEA fraction soluble in ethanolic solution)

Monogalloylglucose with its *m*/*z* 331 [M - H]^−^ ion dissociating to yield an *m*/*z* 169 ion after a glucosyl group loss ([M-H-162]^−^) [39]. Digalloylglucose with its *m*/*z* 483 [M - H]^−^ ion dissociating to yield an *m*/*z* 169 ion after sequential removal of a galloyl group ([M-H-152]^−^) and a glucosyl group ([M-H-162]) [40].

HEEP and HEEA diagnostic mass fragments *m/z* 301 and *m/z* 317 were characterized as quercetin and myricetin, respectively. The neutral losses of 132, 146 and 162 mass units allowed the identification of pentosides (xylose or arabinose), hexosides (glucose or galactose) and deoxyhexoside. Gallic acid was diagnosed by a neutral loss of 152 mass units. Peaks at m/z 433, 447, 463, 593, 609, and 615 were assigned as flavonols and their derivatives. The quercetin pentoside isomer at *m/z* 433 [M – H]^−^ produced the MS/MS fragmentation of *m/z* 300 [M - H-132]^−^, due the loss of arabinopyranoside. The ion at *m/z* 447 was tentatively assigned to quercetin-3-O-β-rhamnose. The MS/MS fragmentation produced a deprotonated aglycone ion at m/z 301 [M-146-H]^−^ due loss a sugar moiety of 146 Da and *m/z* 271 typical of flavon-3-*O*-monoglycoside [41] and 179 from RDA of ring A. Two isomeric compounds ions observed with [M–H]^**−**^ at *m/z* 463, whose MS/MS main fragmentation produced a deprotonated aglycone form myricetin ion at *m/z* 317 [M-146-H]^−^ (loss of a sugar moiety of 146 units), indicates that the compound is a myricetin monohexoside (myricetin 3-O-galactoside or myricetin 3-O-rhamnoside) and another isomer peak ion at *m/z* 301 with its [M-162-H]^−^ (loss of a sugar moiety of 162 units), an indicative of quercetin monohexoside, and the hexose could be glucoside or galactoside [42]. Dissociation of fragment *m/z* 593 showed a loss of 308 units (corresponding to a rhamnose plus glucose group) and yielded directly a fragment ion at *m/z* 285 (assigned as kaempferol).

Compared to flavonoid glycosides found in gingko biloba [43], we tentatively characterized the compound as kaempferol-3-O- glucose- rhamnoside. Fragmentation of the compound at m/z 609 produced an ion at m/z 301, attributed to [M-H-146–162]– through loss of 308 units from a rhamnose (146 u) plus a glucose (162 u), indicating the compound is a rutin.

At *m/z* 615, the MS/MS peak fragmentation produced an ion at *m/z* 463 [M-152-H] (loss of the galloyl moiety) and a deprotonated quercetin at *m/z* 301 [M-162-H]^−^ (loss of a sugar moiety of 162 units), indicative of quercetin-3-O-β-(6”galloyl) hexose. These flavonols derivatives have been previously reported in other *Eugenia* species and they are usually associated to antioxidant and antiproliferative activities [34, 39].

Some phenolic compounds found in HEEP/HEEA extracts belong to the family of ellagitannins. They are hydrolyzable tannins, a class of polyphenols whose structure consists of ellagic acid units linked to a polyol, usually glucose or quinic acid. These compounds are also characterized by their hexahydroxydiphenoyl (HHDP) group which is released on acid hydrolysis and spontaneously lactonizes to ellagic acid. Ellagic acid was characterized by diagnostic mass ion of *m/z* 301 and ma ss fragments at *m*/*z* 257 and 229 [40]. Ellagic acid xyloside was characterized by ion of *m/z* 301[M-162-H] (loss of glucose plus H_2_O, 162 units).

HHDP-glucose isomers were assigned as a signal at *m/z* 481[M-162–18-H] (loss of glucose plus H_2_O, 180 units) [33]. However, ellagitannins had lower efficacy in the inhibition of cell proliferation compared to ellagic acid, the breakdown product [44].

At *m/z* 783, the MS^2^ peak fragmentation produced an ion at *m/z* 481 [M-H-302]^−^, loss of HHDP), and after losing a HHDP-glucose [M–H-481]^−^, an ion at *m/z* 301 which corresponds to ellagic acid. This fragmentation pattern was assigned to a bis-HHDP-glucose isomer. Additionally there were peaks at *m/z* 481 [M-H-469]^−^, loss of a trisgalloyl group) and *m/z* 301, corresponding to ellagic acid. These results suggest a HDDP-glucose and a trigalloyl group. All those results are consistent with data reported for other *Eugenia* [42, 45].

Table 2 shows that EA contains the phenolic compounds myricitrin, rutin, quinic acid and quercetin derivatives. Among those compounds, some present a role on neutrophil activity. Aqueous extract containing quinic acid or its molecule derivate are described to inhibit neutrophil migration [46] and elastase secretion [47]. Despite inhibitory roles demonstrated for myricetin in elastase secretion [48], its derivate myricitrin does not present any inhibitory effect [49]. The suppressor character of rutin on neutrophil functions were better studied than the compounds cited above. Isolated rutin or the one found in plant extracts decreases in vitro and in vivo neutrophil migration [50], adhesion [51], elastase secretion [52] and NET release [27]. Quercetin derivatives were commonly found in *E.aurata* and *E.punicifolia* extract fractions. There are no evidences that quercetin-3-O-β-rhamanose or quercetin-galloyl-hexoside promotes effects on neutrophil activities. In contrast, quercetin suppresses elastase secretion [48] and in vivo and in vitro neutrophil migration [20]. The role of quercetin in the adhesion events is still unclear [53].

ESI-MS^n^ analyses also revealed the presence of gallic acid, quercetin-3-O-α-arabinopyranoside, syringic acid, ellagic acid, monogalloyl-glucose, glycosyringic acid, mudanoside B, HHDP glucose isomer and digalloylglucose isomer. The participation of these compounds in the neutrophil biology is poorly studied. There were reported downregulation of elastase secretion, NET release and neutrophil migration for gallic and ellagic acids present in plant extracts or in isolated form [53]. Evidences were not found for neutrophil adhesion.

## Conclusions

**Fig. 6:**
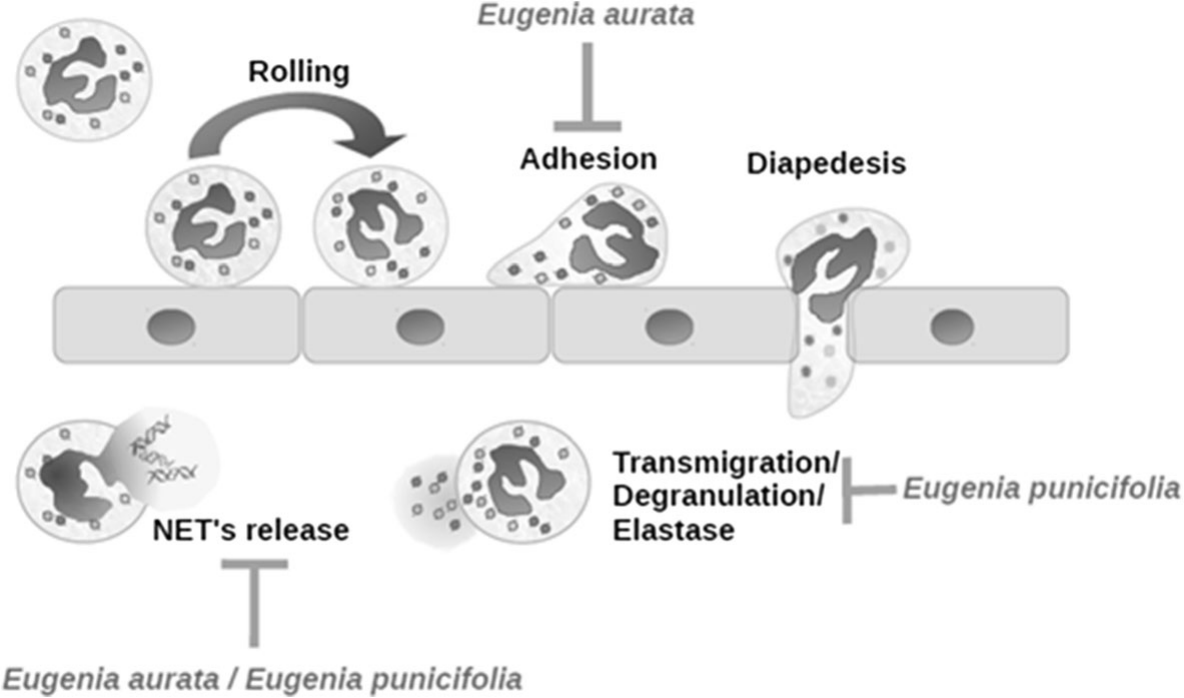
Schematic summary of *Eugenia aurata* and *E. punicifolia* effects on neutrophil recruitment. EA affects mainly adhesion whereas EP, degranulation. Both extracts cease NETs release

Based in our results, we are able to propose a model for the anti-inflammatory properties exhibited by both hydroethanolic extracts of *E. aurata* and *E. punicifolia* (Fig. 6). The extracts in this model have a different chemical composition in terms of phenolic compounds, but both present in vivo and ex vivo anti-inflammatory activity, besides not being toxic to neutrophils. *E. aurata* reduces neutrophil adhesion and *E. punicifolia* decreases elastase degranulation. NET release is inhibited by both extracts ex-vivo. Together, these effects result on a reduced inflammatory response and provide support to their use in popular medicine. Furthermore, these results show a potential of these extracts for the development of phytomedicines with anti-inflammatory properties, including the treatment of rheumatology, neoplastic, self-inflammatory, autoimmune or infectious disorders.

## Abbreviations

DEX: Dexamethasone
DMSO: Dimethyl Sulfoxide
EA: *E. aurata* hydroethanolic extract fraction soluble in PBS
EP: *E.punicifolia* hydroethanolic extract fraction soluble in PBS
ESI-MS: Electrospray ionization mass spectrometry
FBS: Fetal bovine serum
HEEA: *E. aurata* hydroethanolic extract solutions
HEEP: *E. punicifolia* hydroethanolic extract solutions
HPLC: High performance liquid chromatography
MTT: 3-(4,5-dimethylthiazol-2-yl) 2,5-Diphenyl Tetrazolium bromide
NETs: Neutrophil extracellular traps
PBS: Phosphate Buffer Solution
PMA: Phorbol myristate acetate
TG: Thioglycolate
TGI: Total growth inhibition.
